# Effect of second-order magnetic anisotropy on nonlinearity of conductance in CoFeB/MgO/CoFeB magnetic tunnel junction for magnetic sensor devices

**DOI:** 10.1038/s41598-019-53439-0

**Published:** 2019-11-19

**Authors:** Takahiro Ogasawara, Mikihiko Oogane, Muftah Al-Mahdawi, Masakiyo Tsunoda, Yasuo Ando

**Affiliations:** 1 Department of Applied Physics, Tohoku University, Sendai, 980-8579 Japan; 2 Center for Science and Innovation in Spintronics (Core Research Cluster) Organization for Advanced Studies, Tohoku University, Sendai, 980-8577 Japan; 3 Center for Spintronics Research Network, Tohoku University, Sendai, 980-8577 Japan; 4 Department of Electronic Engineering, Tohoku University, Sendai, 980-8579 Japan

**Keywords:** Materials science, Nanoscience and technology

## Abstract

We studied the effect of second-order magnetic anisotropy on the linear conductance output of magnetic tunnel junctions (MTJs) for magnetic-field-sensor applications. Experimentally, CoFeB/MgO/CoFeB-based MTJs were fabricated, and the nonlinearity, NL was evaluated for different thicknesses, *t* of the CoFeB free layer from the conductance. As increasing *t* from 1.5 to 2.0 nm, maximum NL, NL^max^ was found to decrease from 1.86 to 0.17% within the dynamic range, *H*_d_ = 1.0 kOe. For understanding the origin of such NL behavior, a theoretical model based on the Slonczewski model was constructed, wherein the NL was demonstrated to be dependent on both the normalized second-order magnetic anisotropy field of *H*_k2_/|*H*_k_^eff^| and the normalized dynamic range of *H*_d_/|*H*_k_^eff^|. Here, *H*_k_^eff^, *H*_k2_, are the effective and second-order magnetic anisotropy field of the free layer in MTJ. Remarkably, experimental NL^max^ plotted as a function of *H*_k2_/|*H*_k_^eff^| and *H*_d_/|*H*_k_^eff^|, which were measured from FMR technique coincided with the predictions of our model. Based on these experiment and calculation, we conclude that *H*_k2_ is the origin of NL and strongly influences its magnitude. This finding gives us a guideline for understanding NL and pioneers a new prospective for linear-output MTJ sensors to control sensing properties by *H*_k2_.

## Introduction

Magnetic tunnel junctions (MTJs) using a MgO barrier layer have a large tunnel magnetoresistance (TMR) ratio at room temperature^1–6^, and this has made them of interest for a number of spintronic applications such as read heads for hard disk drives and magneto-resistive random access memory. In addition to them, we have used such MTJs for making highly sensitive magnetic-field sensors^7–9^ that can detect very weak bio-magnetic fields^10^. Furthermore, recent industrial progress has increased the variety of uses of magnetic sensors. For instance, electric vehicles (EVs) are equipped with current monitoring systems that have Hall sensors. Here, using MTJ sensors instead of Hall sensors would offer certain advantages: smaller size, lower power consumption^11^ and higher sensitivity^7–9,12^. Magnetic sensors for automobiles must have specific sensing properties: (1) a dynamic range, *H*_d_, over 1 kOe, (2) high sensitivity, and (3) low nonlinearity (NL). Here, *H*_d_ is defined as the range of the magnetic field, *H*, where the sensing properties are evaluated within |*H*| < *H*_d_; for example, *H*_d_ = 1.0 kOe means the sensing properties are evaluated within −1.0 kOe < *H* < 1.0 kOe. Regarding (1), since MTJ sensors are composed of two ferromagnetic electrodes with orthogonal easy axes, the maximum *H*_d_ is determined by the smaller values of the effective anisotropy field, *H*_k_^eff^, of the free layer or the switching field, *H*_sw_, of the pinned layer. Note that the magnetic field is assumed to be applied along the easy axis of the pinned layer. For this reason, utilization of perpendicular magnetic anisotropy (PMA) is especially useful due to the large *H*_sw_ of perpendicularly synthetic antiferromagnetic (p-SAF) coupled Co/Pt multilayers^13–17^ and L1_0_-ordered MnGa alloy^18^. These perpendicular magnetized pinned layers result in a wide dynamic range up to 5.6 kOe^19–22^. Regarding (2), the sensitivity is expressed by the differential coefficient of the TMR curve, which approximately corresponds to the TMR ratio divided by 2|*H*_k_^eff^|. Consequently, a high spin polarization and low |*H*_k_^eff^| are essential for improving sensitivity. Regarding (3), although NL is still not well understood, it is known that its magnitude as evaluated from the resistance, *R*, or conductance, *G*, varies due to its reciprocal relationship. The magnitude of NL evaluated using *G* is found to be smaller than the magnitude evaluated using *R*^21,22^. According to the previous study, a conductance model taking account of only first-order magnetic anisotropy suggests that *G* is expressed by *G* = *G*_0_(1-*P*^2^*H*/*H*_k_^eff^)^21,22^, where *G*_0_ is the conductance at *H* = 0, *P* is the effective spin polarization and *H* is the magnetic field. This equation means that *G* is perfectly proportional to *H*, resulting in NL to be 0 in theory. Although this model can briefly give an interpretation for the smaller NL in *G* than *R*, the finite NL in experiment cannot be explained well. Therefore, for the development of magnetic sensor devices, the origin of NL needs to be understood and its manipulation method should be established.

In this work, we fabricated CoFeB/MgO/CoFeB-based MTJs with p-SAF Co/Pt pinned layers and observed the finite NL which are strongly dependent on CoFeB thickness as well as dynamic range of *H*_d_. In order to analyze the experimental results, we focused on higher order magnetic anisotropy of *H*_k2_ of the free layer and include its effect in the conductance calculation using the Slonczewski model^23^ with simultaneous magnetization rotation. The calculation suggests that the maximum NL, NL^max^, decreases as the normalized second-order anisotropy, *H*_k2_/|*H*_k_^eff^|, and the normalized dynamic range, *H*_d_/|*H*_k_^eff^|, decrease. Based on these experimental and theoretical results, the origin and the controlling method of NL will be discussed.

## Experimental Method

All the samples were deposited on Si/SiO_2_ substrates at room temperature by using dc/rf magnetron sputtering with a base pressure less than 1.0 × 10^–6^ Pa. The stacking structure of the MTJs were Ta(3)/Ru(10)/Pt(2)/[Co(0.28)/Pt(0.16)]_9_/Co(0.28)/Ru(0.4)/Co(0.28)/[Pt(0.16)/Co(0.28)]_5_/Ta(0.2)/Co_40_Fe_40_B_20_(1.0)/MgO(2)/Co_20_Fe_60_B_20_(*t*)/Ta(5)/Ru(8) (thickness in nm). The thickness of the Co_20_Fe_60_B_20_ free layers, *t* was varied from 1.5 to 2.0 nm. After patterning the samples into circular junctions with diameters of 100 µm by photolithography and Ar ion milling, the MTJs were post-annealed at 300 °C in a vacuum furnace. TMR curves were measured using the dc-four-probe method. To evaluate *H*_k_^eff^ and *H*_k2_, we carried out angle-dependent ferromagnetic resonance (FMR) on samples consisting of Ta(3)/MgO(2)/Co_20_Fe_60_B_20_(*t*)/Ta(5), which corresponds to the free layer of the MTJs. Microwaves with a frequency of 9.4 GHz (X-band) were applied to the TE_011_ cavity holding the sample, and the resonance spectra were lock-in detected. The magnetization curves were measured with a vibrating sample magnetometer (VSM). All measurements were carried out at room temperature.

## Experimental Results

**Figure 1:**
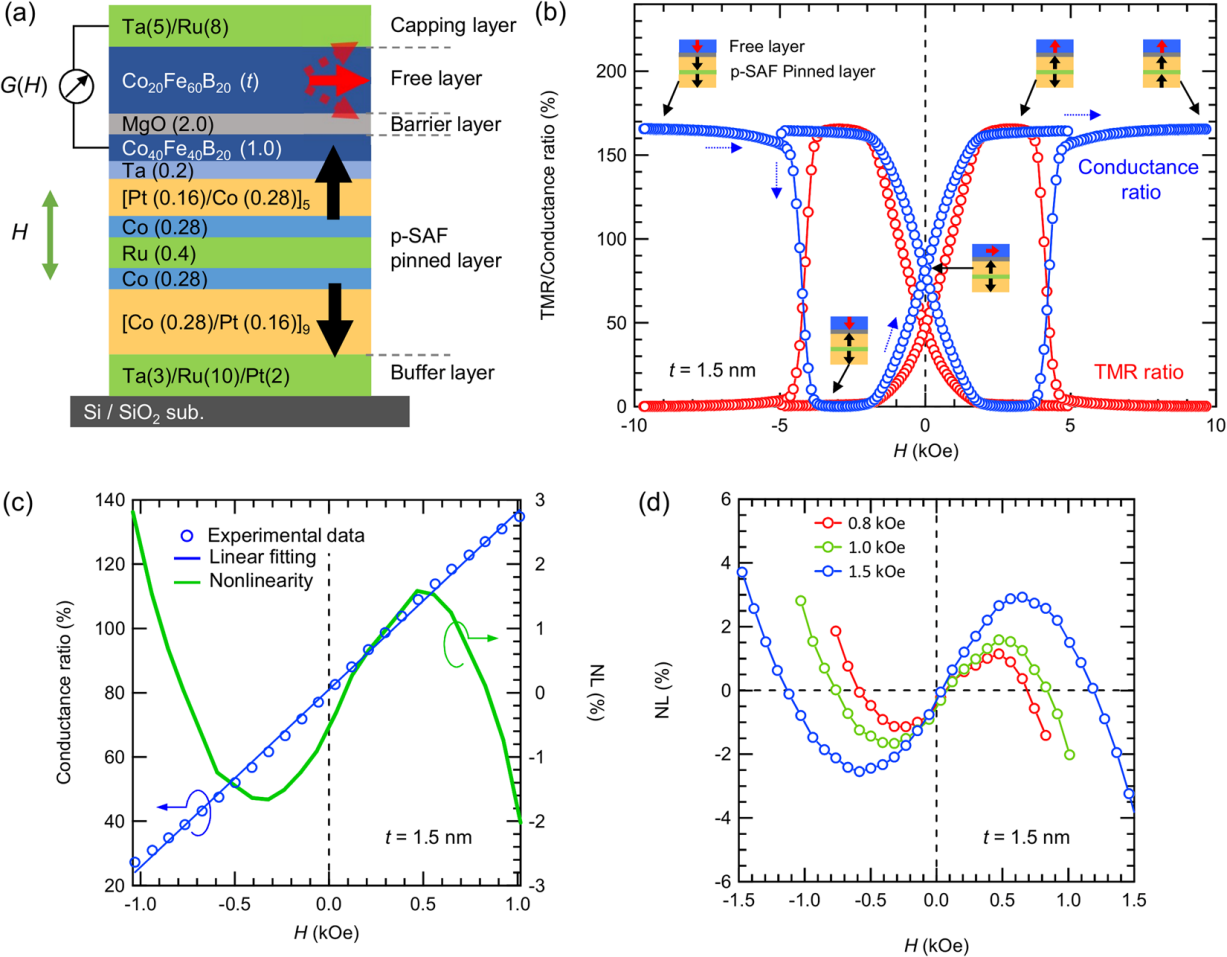
(**a**) The schematic of CoFeB/MgO/CoFeB-MTJ using p-SAF coupled [Co/Pt] multilayer via Ru for pinned layer. (**b**) Full scale of TMR and conductance ratio curves as a function of *H* (red and blue circles, respectively) for 1.5-nm-thick CoFeB free layer. The schematic diagrams show a MTJ consisting of a free layer and p-SAF pinned layers together with the expected magnetization directions for conductance ratio curve under the magnetic field swept from negative to positive. The blue dotted arrows indicate the transition of the conductance ratio curves from negative to positive magnetic field. (**c**) Conductance ratio within the range of ±1.0 kOe and the NL curve from negative to positive magnetic field. The blue circles and line are experimental and linear fitted data within *H*_d_ = 1.0 kOe, respectively. The green line is the corresponding NL curve. (**d**) NL curves for different *H*_d_ of 0.8,1.0 and 1.5 kOe for 1.5-nm-thick CoFeB free layer from negative to positive magnetic field.

**Figure 2:**
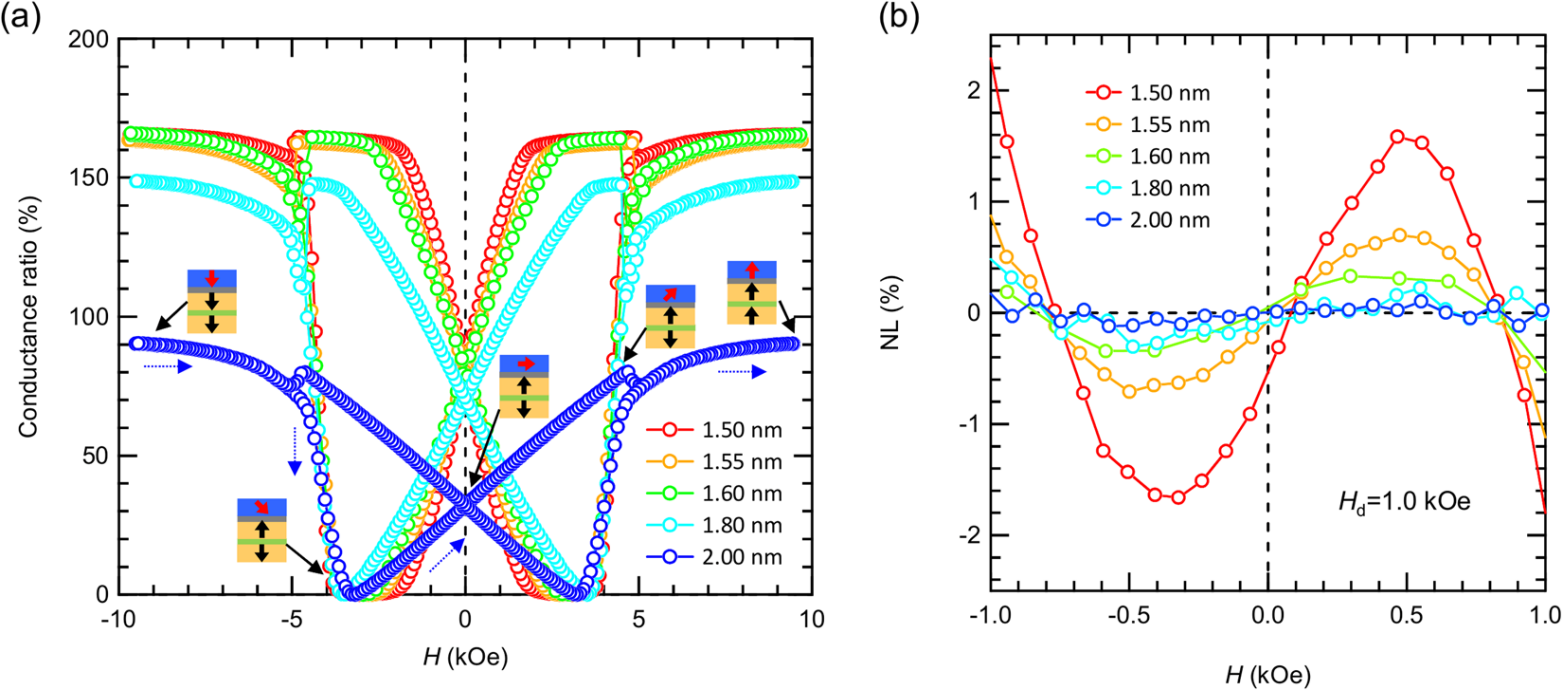
(**a**) Full scale conductance ratio curves for *t* = 1.5–2.0 nm. The schematic diagrams show an expected magnetization in MTJ with 2.0-nm-thick CoFeB under the magnetic field from negative to positive. The blue dotted arrows indicate the transition of the conductance ratio curves from negative to positive magnetic field. (**b**) NL curves for *t* = 1.5 to 2.0 nm from negative to positive magnetic field.

Firstly, let us examine the experimentally measured conductances of the MTJs. As shown in Fig. 1(a) depicting the schematic of our fabricated MTJ, we employed the p-SAF Co/Pt multilayers for the pinned layer of MTJ because our Co/Pt multilayer shows large PMA of c.a. 5 Merg/cm^3^ and can be coupled via thin Ru spacer, resulting in the strong antiferromagnetic coupling^17^. Therefore, this magnetization robustness against magnetic field due to the strong coupling is favorable for NL measurements in wide range of *H*_d_^21^. Figure 1(b) shows the TMR and conductance ratio for an MTJ with a 1.50-nm-thick CoFeB free layer under the perpendicular magnetic field. The TMR ratio is defined using a typical expression as shown in Eq. (1), which is normalized by the parallel resistance, *R*_P_. On the other hand, since some of our MTJs lack anti-parallel state due to largely negative *H*_k_^eff^ depending on the thickness (see Fig. 2(a)), the conductance ratio are normalized as in Eq. (2) using minimum conductance of *G*_min_. However, it should be noted that the magnitude of TMR and conductance ratio can match each other.

1 $$\begin{array}{c}{\rm{TMR}}\,{\rm{ratio}}=\frac{R(H)-{R}_{{\rm{P}}}}{{R}_{{\rm{P}}}}\,\times 100\,( \% )\end{array}$$

2 $$\begin{array}{c}{\rm{Conductance}}\,{\rm{ratio}}=\frac{G(H)-{G}_{\min }}{{G}_{\min }}\times 100\,( \% )\end{array}$$

The results in the Fig. 1(b) show a large TMR ratio, c.a. 166%, even for post-annealing at 300 °C. This is thought to be due to our optimum structure of MTJs with a flat surface^17^. The TMR and the conductance curve show opposite trends against *H*, because they are in reciprocal relationship with one another, but the magnitudes of TMR and the conductance ratio accord with each other. Taking the conductance ratio curve of Fig. 1(b), for example, the large dip at c.a. ±4 kOe is due to magnetization reversal of the upper [Co/Pt] layer of the p-SAF pinned layer for the antiferromagnetic coupling which corresponds to *H*_sw_. The rotation of the magnetization of the free layer appears within c.a. ±1.8 kOe which corresponds to |*H*_k_^eff^| of the free layer. Notably, the *H* dependence of the TMR and conductance curves are different within ±1.8 kOe. Since *G* (*R*) is proportional (inversely proportional) to cos*θ* of the free layer from the Slonczewski model^24^, where *θ* is the relative angle of magnetizations, the curves have significantly different shapes where the free layer rotates^21,22^. This contributes to the NL determined from *G* being smaller than the NL determined from *R*. Figure 1(c) shows the nonlinearity for 1.5-nm-thick CoFeB determined from the conductance ratio at *H*_d_ = 1.0 kOe using Eq. (3), where *G* is the conductance, *G*_fit_ is the linear fitting to *G*, and *G*_max(min)_ is the maximum (minimum) *G* within the range of *H*_d_.

3 $$\begin{array}{c}{\rm{NL}}=\frac{G-{G}_{{\rm{fit}}}}{{G}_{{\rm{\max }}}-{G}_{{\rm{\min }}}}\times 100\,( \% )\end{array}$$

This equation quantifies the normalized differences between conductance and its linear fitting and consequently gives *H*-dependent NL curves. Since NL is expressed by removing a linear component of the fitting from *G*, the shape of *G* can be emphasized in *H*-dependent NL. As shown in Fig. 1(c), slight difference between *G* and *G*_fit_ are seen, which gives finite S-shaped NL. Also, we show the *H*_d_ dependence of NL for this sample in Fig. 1(d). As increasing *H*_d_ from 0.8 to 1.5 kOe, the magnitude of NL increases. Here, the absolute maximum of NL is defined as NL^max^ and it changes from 1.86 to 3.82% in that range. This is due to the increasing of (*G* − *G*_fit_) as expanding *H*_d_ which means that the difference between experiment and linear fitting is more incorporated by evaluating NL in the larger range of magnetic field.

**Figure 3:**
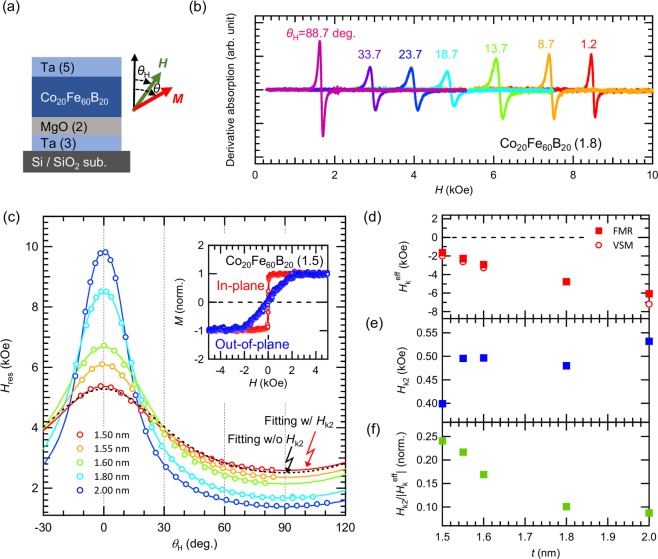
(**a**) Schematic of the sample with MgO/CoFeB/Ta and coordinate system for FMR measurement and analysis. (**b**) Typical FMR spectra for 1.8-nm-thick CoFeB at various magnetic field angle. (**c**) Angular dependent *H*_res_ data for Ta/MgO/CoFeB (1.5–2.0)/Ta films. Circles and solid lines are experiment and fitting data with *H*_k2_, respectively. The black dotted line is the example of the fitting without *H*_k2_ for 1.5-nm-thick CoFeB. The angles *θ*_H_ = 0 and 90 degree correspond to the out-of-plane and in-plane magnetic field from the sample plane. Also, the inset shows the typical magnetization curve of 1.5-nm-thick CoFeB. (**d–f**) Thickness dependence of *H*_k_^eff^, *H*_k2_ and *H*_k2_/|*H*_k_^eff^|.

Figure 2(a) shows the free layer thickness dependence of the conductance ratio of the MTJ with perpendicular magnetic field. For thicker sample, the conductance ratio decreases compared to thinner samples. As increasing *t*, the *H*_k_^eff^ of free layer negatively increase because the demagnetizing field becomes more dominant than the interfacial magnetic anisotropy. Since the magnetization of the free layer in MTJ starts to rotate at *H* ≈ *H*_k_^eff^ from negative to positive magnetic field, an anti-parallel state cannot be seen in MTJ with negatively large *H*_k_^eff^. For example, the schematic of Fig. 2(a) shows the expected magnetization of MTJ with 2.0-nm-thick CoFeB free layer. As a 2.0-nm-thick CoFeB exhibits the *H*_k_^eff^=−6.2 kOe (discussed later in Fig. 3(d)), the anti-parallel state becomes absent under the region of the pinned layer showing antiferromagnetic coupling at around |*H*|* < *4 kOe. This is the reason for the thickness dependence of the magnitude of the conductance ratio. However, the linear *G* outputs can be obtained in all samples in the vicinity of *H* = 0. For these samples, NL is evaluated at *H*_d_ = 1.0 kOe and summarized in Fig. 2(b). This graph shows that NL is highly dependent upon free layer CoFeB thickness. As increasing *t* from 1.5 to 2.0 nm, NL^max^ decreases from 1.86 to 0.17%. Therefore, it is found that a highly linear *G* output can be achieved by increasing the thickness, however, this thickness controlling method is not favorable since sensitivity (~TMR ratio/2|*H*_k_^eff^|) decreases due to negatively larger *H*_k_^eff^ in thick CoFeB. By summarizing our experiments above, NL are strongly dependent on *H*_d_ and CoFeB thickness. However, according to the previous conductance model of *G* = *G*_0_(1-*P*^2^*H*/*H*_k_^eff^) with taking only first magnetic anisotropy^21,22^, *G* is completely linear to *H*, where NL is theoretically expected to be zero in all range of *H*_d_ and all CoFeB thickness. In order to find clues for understanding the origin and behavior of NL, we characterized the magnetic properties of CoFeB thin films by means of FMR.

Figure 3(a) shows schematic of the sample of MgO/CoFeB/Ta stacks, which corresponds to the free layer of our MTJ and the coordinate system for FMR measurements. For the FMR analysis, the resonance condition based on the Landau-Lifshitz-Gilbert equation can be written as Eqs (4–6), where *f* is the microwave frequency, *γ* the gyromagnetic ratio, and *H*_res_ the resonance field^24,25^. From the angular-dependent *H*_res_, the experimental data are fitted by Eqs (4–6 and (8), which in turn give magnetic properties such as *H*_k_^eff^ and *H*_k2_.

4 $$\begin{array}{c}f=\frac{\gamma }{2\pi }\sqrt{{H}_{1}{H}_{2}}\end{array}$$

5 $$\begin{array}{c}{H}_{1}={H}_{{\rm{res}}}\,\cos (\theta -{\theta }_{{\rm{H}}})+{H}_{{\rm{k}}}^{{\rm{eff}}}{\cos }^{2}\theta -{H}_{{\rm{k}}2}{\cos }^{4}\theta \end{array}$$

6 $$\begin{array}{c}{H}_{2}={H}_{{\rm{res}}}\,\cos (\theta -{\theta }_{{\rm{H}}})+{H}_{{\rm{k}}}^{{\rm{eff}}}\,\cos \,2\theta \,-\frac{1}{2}{H}_{{\rm{k}}2}(\cos \,2\theta +\,\cos \,4\theta )\end{array}$$

For Eq. (7), the magnetic energy density per unit volume for the free layer, *E*, is well described by summing the Zeeman energy, demagnetizing energy, and magnetic anisotropy. Here, *M*_s_ is the saturation magnetization, *H* and *θ*_H_ are the magnetic field and its angle, and *K*_1_ and *K*_2_ are the first- and second-order uniaxial magnetic anisotropy constants, respectively. Also, by the deformation of *E* as in Eq. (7), we define the coefficient of cos^2^*θ* as an effective first-order magnetic anisotropy, *K*_1_^eff^ which is equal to *K*_1_ + 2*K*_2_ − 2π*M*_s_^2^. Since the magnetization angle of the free layer follows d*E*/d*θ* = 0, magnetization angle is determined by the first derivative of *E* described in Eq. (8), where *H*_k_^eff^ is the effective first-order anisotropy field given by 2*K*_1_/*M*_s_ + 4*K*_2_/*M*_s_−4π*M*_s_ and *H*_k2_ is the second-order anisotropy field given by 4*K*_2_/*M*_s_.

7 $$\begin{array}{c}E=-{M}_{s}H\,\cos ({\theta }_{H}-\theta )+{K}_{1}{\sin }^{2}\theta +{K}_{2}{\sin }^{4}\theta +2\pi {M}_{s}^{2}{\cos }^{2}\theta \,\\ \,=-{M}_{s}H\,\cos ({\theta }_{H}-\theta )-{K}_{1}^{{\rm{eff}}}{\cos }^{2}\theta +{K}_{2}{\cos }^{4}\theta +{K}_{1}+{K}_{2}\end{array}$$

8 $$\begin{array}{c}\sin \,2\theta =\frac{2H}{{H}_{{\rm{k}}}^{{\rm{eff}}}}\,\sin ({\theta }_{H}-\theta )+\frac{{H}_{{\rm{k}}2}}{{H}_{{\rm{k}}}^{{\rm{eff}}}}\,\sin \,2\theta {\cos }^{2}\theta \end{array}$$

Figure 3(b) shows typical FMR spectra for 1.8-nm-thick CoFeB at various magnetic field angle. The observed spectra are Lorentzian-like shapes with peak-to-peak line width of several hundred Oe, which is typical for MgO/CoFeB/Ta film due to the spin-pumping effect^25,26^. Figure 3(c) shows the angle-dependent *H*_res_ for *t* = 1.5–2.0 nm. For all thicknesses, the minimum *H*_res_ occurs at *θ*_H_ = 90 deg., which indicates that all samples exhibit in-plane magnetic anisotropy. The solid lines in Fig. 3(c) are fittings using Eqs (4–6 and (8) with incorporate the effect of *H*_k2_ to the experimental data and show a good coincidence. It should be noted that if the data is fitted without *H*_k2_ (i.e. *H*_k2_ = 0), as shown in a black dotted line in Fig. 3(c) for 1.5-nm-thick CoFeB as an example, the differences between the experiment and the fitting becomes larger. Hence, the effect of *H*_k2_ in our films is not negligible. The best fitting parameter for 1.5-nm-thick CoFeB from FMR is determined as *H*_k_^eff^ = −1.7 kOe and *H*_k2_ = 0.4 kOe, which approximately match the results of magnetization curve, displayed in the inset of Fig. 3(c). For the thickness dependence of *H*_res_, as *t* increases, *H*_res_ at *θ*_H_ = 0 deg. increases and that at *θ*_H_ = 90 deg. decreases, which suggests that thicker CoFeB film has a negatively larger *H*_k_^eff^ (i.e., larger in-plane magnetic anisotropy). Figure 3(d–f) summarizes the magnetic properties of *H*_k_^eff^ and *H*_k2_ versus CoFeB thickness as given by the FMR fittings using Eqs (4)-(6) and (8). Also, *H*_k_^eff^ obtained from the magnetization curves from VSM are shown in Fig. 3(d) as the reference. Figure 3(d) shows that *H*_k_^eff^ increases as the CoFeB layer gets thinner, which is due to the presence of a well-defined interfacial magnetic anisotropy^6,27–29^. On the other hand, *H*_k2_ shows the opposite trend; that is, *H*_k2_ decreases as *t* decreases. The reason for this *H*_k2_ dependence is not clear at present, but there may be effects from the interfacial magnetic anisotropy, even for *H*_k2_ similarly to *H*_k_^eff^, since *H*_k2_ has been reported to depend on the thickness of the CoFeB layer^30^. Some studies have indicated that CoFeB strain and/or surface roughness may increase the magnitude of higher order magnetic anisotropy^31,32^. We consider that the structural and surface properties of CoFeB may vary with the thickness, because CoFeB begins to crystallize from the contacted MgO layer by solid-phase epitaxy and this causes a mixture of bcc and amorphous textures with different interface structures^33^. Therefore, we can infer that, as pointed out above, the effects of the interfacial anisotropy, crystallinity, and interfacial condition are reflected to some extent in *H*_k2_ of CoFeB films. Figure 3(f) plots *H*_k2_/|*H*_k_^eff^| as a function of *t*, where FMR fitting results of *H*_k_^eff^ and *H*_k2_ in Fig. 3(d,e) are used. Remarkably, in our samples, although the ratio of *H*_k2_/|*H*_k_^eff^| is moderate at approximately 0.01 for thicker CoFeB, it increases as decreasing *t*, resulting in the maximum of *H*_k2_/|*H*_k_^eff^| = 0.24 for *t* = 1.50 nm. This result quantitatively suggests that the effect of *H*_k2_ cannot be negligible for thin CoFeB film. As shown in Eq. (8), since the second-order anisotropy gives additional term of *θ* dependence for the magnetization rotation, this *H*_k2_ term is expected to bring some minor change for the conductance curve compared to the model using only first order magnetic anisotropy of *G* = *G*_0_(1-*P*^2^*H*/*H*_k_^eff^)^21,22^. Therefore, second-order magnetic anisotropy possibly is expected to give rise to the finite NL.

## Model Calculation

Next, let us discuss the effect of second-order magnetic anisotropy on the conductance curves. For the tunnel conductance calculation, we used a Slonczewski model where electrons are transmitted through a rectangular barrier potential. This model can express the TMR phenomena well^23^. The conductance, *G*, follows Eq. (9), where *θ* is the angle of the free-layer ferromagnet, measured from the direction normal to the film plane, *P* is the effective spin polarization and *G*_0_ is the conductance at *θ* = π/2.

9 $$\begin{array}{c}G={G}_{0}(1+{P}^{2}\,\cos \,\theta )\end{array}$$

**Figure 4:**
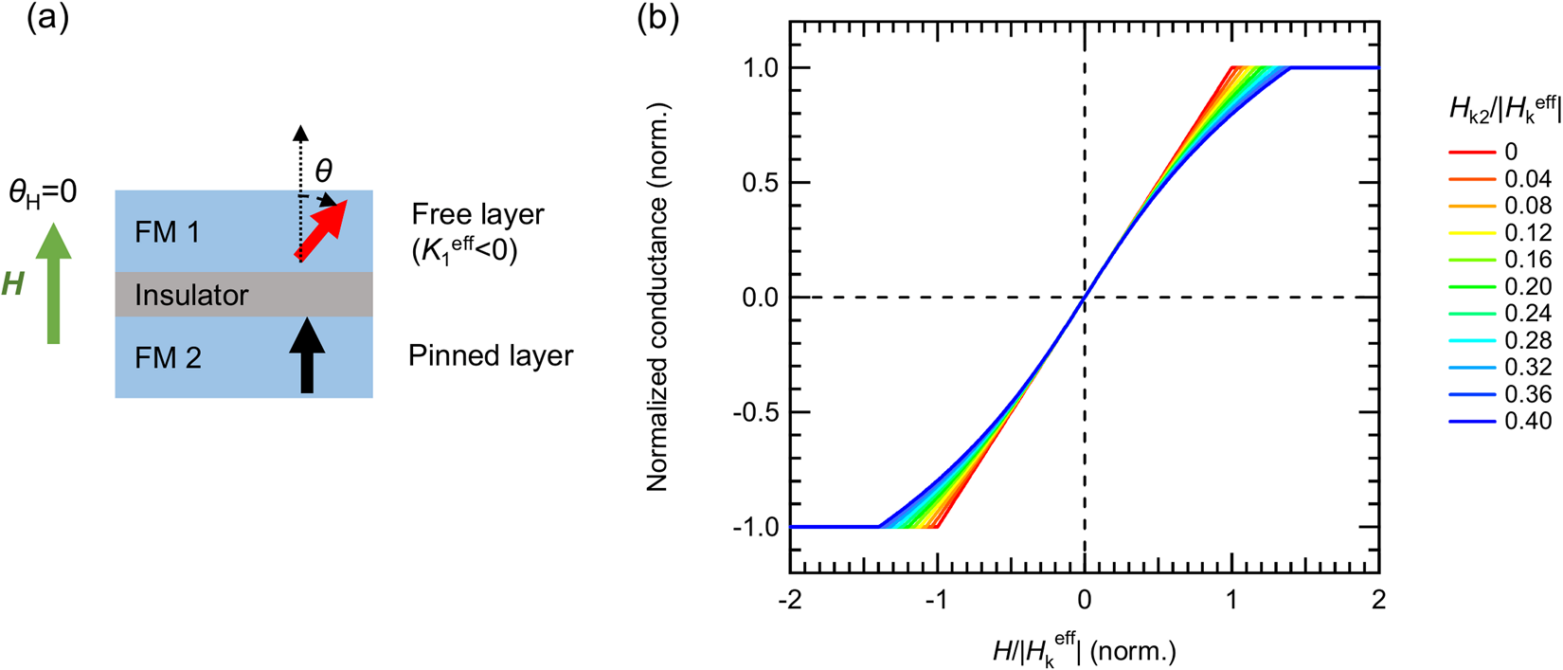
(**a**) Schematic diagram of MTJ consisting of two ferromagnetic layers, FM1 and FM2, with an insulating layer between them. FM1 and FM2 are the in-plane magnetized free layer and perpendicularly magnetized pinned layer, respectively. We assume that the magnetization of FM2 and *H* is fixed along perpendicular to the film plane. (**b**) Normalized conductance of (*G* − *G*_0_)/*G*_0_*P*^2^ as a function of *H*/|*H*_k_^eff^| for various magnitudes of *H*_k2_/|*H*_k_^eff^|.

As shown in Fig. 4(a), we assume that the magnetization direction of pinned layer is fixed along the direction perpendicular to the film plane. We also employed a simultaneous rotation model where the magnetization direction rotates following the absolute minimum of the magnetic energy. This means that the first and second derivatives of *E* of the free layer are in the condition of d*E*/d*θ* = 0 (Eq. (8)) and d^2^*E*/d*θ*^2^ > 0. Here, the equation d*E*/d*θ* = 0 can be simplified to Eq. (10) under a perpendicular magnetic field with *θ* ≠ 0 or π.

10 $$\begin{array}{c}\cos \,\theta =-\,\frac{H}{{H}_{{\rm{k}}}^{{\rm{eff}}}}+\frac{{H}_{{\rm{k}}2}}{{H}_{{\rm{k}}}^{{\rm{eff}}}}{\cos }^{3}\theta \,({\theta }_{H}=0,\,\theta \ne 0,\,\pi )\end{array}$$

From Eqs (9) and (10), it is clear that once *H*_k2_ is 0, *G* is directly proportional to *H*. However, a finite value of *H*_k2_ causes minor changes to the shape of the conductance curves. Here, in Fig. 4(b), we plotted the normalized conductance of (*G* − *G*_0_)/*G*_0_*P*^2^ against the normalized magnetic field, *H*/|*H*_k_^eff^|, for different *H*_k2_/|*H*_k_^eff^|. The Fig. 4(b) confirms that as *H*_k2_/|*H*_k_^eff^| increases, the conductance curves distinctively change the shapes as expected from Eqs (9) and (10). This is due to the second-order magnetic anisotropy effect.

**Figure 5:**
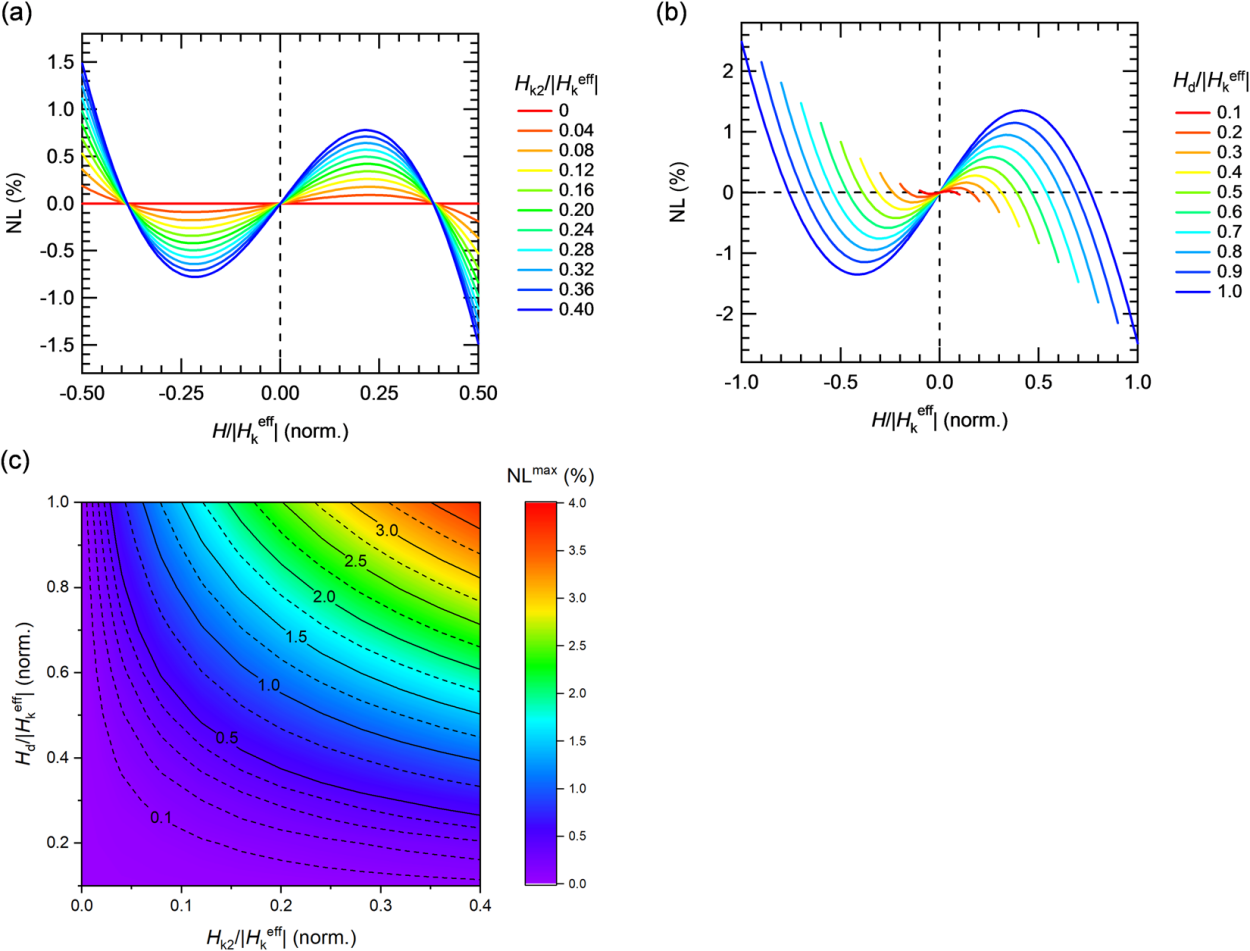
(**a**) NL curves with *H*_d_/|*H*_k_^eff^| = 0.50 and *H*_k2_/*H*_k_^eff^ variations. (**b**) NL curves with *H*_k2_/|*H*_k_^eff^| = 0.2 with *H*_d_/|*H*_k_^eff^| variations. (**c**) Summary of NL^max^ as a function of *H*_k2_/|*H*_k_^eff^| and *H*_d_/|*H*_k_^eff^|.

Figure 5(a) shows the NL curves determined from the conductance curves in Fig. 4(b) by using Eq. (3) for different normalized second-order magnetic anisotropy, *H*_k2_/|*H*_k_^eff^| within the normalized dynamic range *H*_d_/|*H*_k_^eff^| = 0.5. We found that similar S-shaped NL curves observed in our experiment are reproduced in the calculation under *H*_k2_ ≠ 0 and it changes to 0 for all range of *H*/|*H*_k_^eff^| under *H*_k2_ = 0. Although the shape of NL remains almost unchanged, the magnitude of NL obviously decreases as *H*_k2_/|*H*_k_^eff^| decreases. This calculated result can give an explanation of experimental thickness dependence of NL (see Fig. 2(b)) which is linked to the magnitude of *H*_k2_/|*H*_k_^eff^| (see Fig. 3(f)). Hence, we conclude from these calculations that the observed S-shaped NL curves and the magnitudes originate from the presence of *H*_k2_. Additionally, NL can be scaled by the magnitude of *H*_d_/|*H*_k_^eff^|. As shown in Fig. 4(b), *G* is extremely linear in a very small magnetic field, but not in large magnetic field under *H*_k2_/|*H*_k_^eff^| ≠ 0. The NL curves with different *H*_d_/|*H*_k_^eff^| under *H*_k2_/|*H*_k_^eff^| = 0.2 are shown in Fig. 5(b). As expected, NL increases with *H*_d_/|*H*_k_^eff^| due to the curving effect in the large magnetic field from the *H*_k2_ term. Additionally, this model can briefly explain our results showing an NL^max^ increase in a large dynamic range (see Fig. 1(d)). Figure 5(c) summarizes the calculated NL^max^ against the variation in *H*_k2_/|*H*_k_^eff^| and *H*_d_/|*H*_k_^eff^|. As |*H*_k_^eff^| increases, both *H*_k2_/|*H*_k_^eff^| and *H*_d_/|*H*_k_^eff^| decrease, which results in a small NL. However, the reduction in NL by increasing |*H*_k_^eff^| is in a trade-off relationship with the sensitivity, as mentioned above. In our model that considers second-order magnetic anisotropy, NL decreases with decreasing *H*_k2_ for all values of *H*_d_/|*H*_k_^eff^|, without the sensitivity deteriorating. Therefore, *H*_k2_ in the free layer plays an important role and this model gives us a guideline for designing high-performance MTJ sensors.

**Figure 6:**
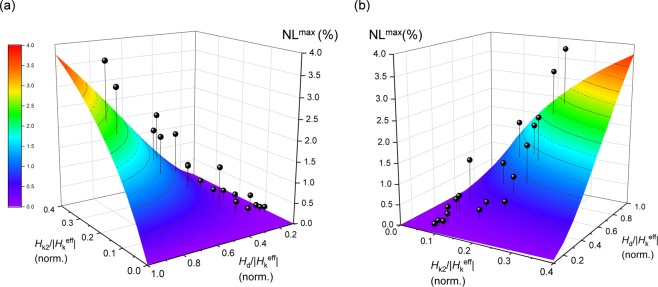
(**a,b**) NL^max^ as a function of *H*_k2_/|*H*_k_^eff^| and *H*_d_/|*H*_k_^eff^| from different views. The surface and points are calculated and experimental data.

Consequently, we investigated the correspondence between the experimental and calculated values of NL^max^ for our MTJs with different CoFeB thicknesses of 1.5–2.0 nm which corresponds to the variation of *H*_k2_/|*H*_k_^eff^| of 0.09–0.24 and different *H*_d_ of 0.8, 1.0, 1.5 and 2.0 kOe which corresponds to the variation of *H*_d_/|*H*_k_^eff^| of 0.13–0.89. Note that *H*_d_ is limited up to 2.0 kOe in order to ensure the magnetic field range with well-fixed pinned layer for NL evaluation. Figure 6(a,b) summarizes NL^max^ as a function of *H*_k2_/|*H*_k_^eff^| and *H*_d_/|*H*_k_^eff^|. The experimental results approximately coincide well with the calculations, that is, as decreasing *H*_k2_/|*H*_k_^eff^| and *H*_d_/|*H*_k_^eff^|, experimental results of NL^max^ decreases. Although a slight discrepancy can be seen resulting in larger NL^max^ which might be due to experimental error or other effects that are not considered in the calculation, such as a slight fluctuations (not a flip but a rotation in microscopic range of the angle) of the pinned layer, angular dispersion, other higher anisotropy terms, and/or tunnel anisotropic magnetoresistance (TAMR) effect, we can approximately explain the NL^max^ trend by *H*_k2_/|*H*_k_^eff^| and *H*_d_/|*H*_k_^eff^|. The details of these minor influence on NL should be the studied more, however, we conclude that predominantly both of *H*_k2_/|*H*_k_^eff^| and *H*_d_/|*H*_k_^eff^| are intrinsic for NL control.

## Conclusion

In conclusion, we studied the effect of second-order magnetic anisotropy on the linearity of the output in MTJ sensors. In experiment, we fabricated CoFeB/MgO/CoFeB-MTJs and S-shaped NL curve are observed in all samples. In addition, for the magnitude of NL, a clear *H*_d_ and thickness dependence is found. In order to investigate the origin of NL, we calculated the NL using Slonczewski model with incorporating the effect of second-order magnetic anisotropy. From the calculation, S-shaped NL curve is reproduced under *H*_k2_ ≠ 0 and NL^max^ is found to be strongly dependent on both of *H*_k2_/|*H*_k_^eff^| and *H*_d_/|*H*_k_^eff^|. Remarkably, experimental and calculated NL^max^ are in a good agreement, therefore, we conclude that both of *H*_k2_/|*H*_k_^eff^| and *H*_d_/|*H*_k_^eff^| are intrinsic for NL in MTJ. Thus, this study provides an understanding for the phenomena of NL and pioneers the new method to control sensing properties of MTJ by second-order anisotropy.
